# The Potential Risk of Nanoparticulate Release from Photocatalytic Pavement Concrete Surface Due to a Simulated Abrasion Load—An Experimental Study

**DOI:** 10.3390/ma17123022

**Published:** 2024-06-20

**Authors:** Hubert Witkowski, Janusz Jarosławski, Artur Szkop, Karol Chilmon, Maciej Kalinowski, Wioletta Jackiewicz-Rek

**Affiliations:** 1 Faculty of Civil Engineering, Warsaw University of Technology, 00-637 Warsaw, Poland; karol.chilmon@pw.edu.pl (K.C.); maciej.kalinowski@pw.edu.pl (M.K.); wioletta.rek@pw.edu.pl (W.J.-R.); 2 Institute of Geophysics, Polish Academy of Sciences, 01-452 Warsaw, Poland; januszj@igf.edu.pl (J.J.); aszkop@igf.edu.pl (A.S.)

**Keywords:** photocatalytic cementitious composites, abrasion, nanoparticulate emissions, photocatalytic nanomaterials, TiO_2_

## Abstract

The risk of the releasing of nanometric particles from construction materials with nanometric components might be one of the biggest threats to further development of them. One of the possible ingress routes to human organisms is the respiratory system. Therefore, it is crucial to determine the risk of emission of nanometric particles during material usage. In the presented paper, abrasion of mortar samples with nanometric TiO_2_ was investigated. A special abrasion test setup was developed to reflect everyday abrasion of the concrete surface of pavements. In the study, three TiO_2_-modifed mortar series (and respective reference series) underwent the developed test protocol and the grains were mobilized from their surface due to the applied load analyzed (granulation, morphology, and chemical composition). For a comparative analysis, an abrasion parameter was developed. Based on the obtained results, the modification of cementitious composites with nanometric TiO_2_ contributed to a reduction in the emission of aerosols and, therefore, confirmed the compatibility between TiO_2_ and cement matrix.

## 1. Introduction

The use of nanometric components in building materials has completely revolutionized building materials in recent decades. Materials at atomic and molecular scales exhibit significantly different properties from those at a larger scale [1,2,3,4]. Many laboratories and academic centers research the modification of cement materials using nanomaterials such as nano-clays, nanoCaCO_3_, and nanoSiO_2_ [5]. Through this type of modification, the mechanical properties of cementitious materials can be enhanced, the use of Portland cement clinker in total binder mass can be reduced, or new and desired properties can be added to concrete’s characteristics. One of the latter is the potential to improve environmental conditions (reduce the concentration of gaseous pollutants—both organic and inorganic) due to the inclusion of nanoparticulate photocatalytic materials in the composition of cementitious composite. Such composites have self-cleaning properties and can reduce the concentration of airborne pollution through photocatalytic reactions [6]. The most investigated photocatalyst in the construction industry has been titanium dioxide (TiO_2_). Photocatalytic cementitious materials with nanometric TiO_2_ have already been used in many civil and building applications, such as tunnels [7,8,9], concrete facade elements [10,11,12,13], and concrete pavements [14,15,16,17,18].

Once irradiated with an electromagnetic wave of specific characteristics, electrons from nanoparticulate photocatalysts (semiconductor–titanium oxide, zinc oxide, etc.) shift bands from valance to conduction, forming electron holes. This phenomenon initiates various reduction–oxidation processes caused by the creation of hydroxyl radicals and superoxide radicals, allowing for the decomposition/conversion of various gaseous pollutants [19]. Considering the use of photocatalytic cementitious composites as a pavement material for roads, sidewalks, and other surface types exposed to solar radiation, their main potential lies in reducing the photochemical pollution caused by urban traffic—mainly nitrogen oxides, near-surface ozone, and aromatic hydrocarbons [6,20]. As the aforementioned types of pollution cause significant risks to human health and contribute to climate change acceleration, using building materials with functions to passively reduce their concentration over large areas is justified and desirable. However, it is essential to note that their use cannot cause an increase in other types of pollution.

Nanomaterials are widely used in various areas of everyday life—nanometric TiO_2_ can be found, among others, in cosmetics, toothpaste, suncream [21], and even food [22]. It has been proven that exposure to a nanoparticulate TiO_2_ increases the risk of adverse effects on human health, such as pulmonary inflammation and increased risk of many diseases, tumors, or the progress of existing cancer processes [23]. The average crystallite size of photocatalytic materials used in the construction sector is smaller than 20 nm [24]. As crystallites usually agglomerate, the average diameter of such agglomerated grains is usually below 800 nm. That being said, the nanoscale dimensions of such grains could be treated as suspended dust, categorized as atmospheric aerosols with a maximum diameter of 2.5 μm, considered the most dangerous to human health. Such aerosols could enter and accumulate within the human respiratory system, possibly causing harm in the case of long-lasting exposure [25]. The health impact of nanomaterials on human health is still under investigation. Studies on the effects of TiO_2_ nanoparticles on the pulmonary system show both local and systemic effects and intensified pre-existing symptoms.

Dylla and Hassan [26] investigated the issue of nanoparticulate emissions from photocatalytic pavements during construction activity. The study measured the size distribution of nanoparticles released during laboratory and field activity for photocatalytic mortar overlays and photocatalytic spray coatings. The obtained nanoparticle counts and size distribution curves suggested that TiO_2_ nanoparticles were released when applying photocatalytic pavements. However, identification of the nanoparticles was not possible due to difficulties in obtaining high-resolution images. Bossa et al. [27] investigated nanometric TiO_2_ release from photocatalytic cement paste in a static leaching test. After 168 h of leaching, less than 0.04 w.% of the initial nanometric TiO_2_ had been released from the cement pellets. Boonen et al. [28] investigated two types of abrasion on photocatalytic paving elements and surface coatings. The coating durability was tested according to the abrasion test for glazed ceramic tiles following NBN EN ISO 10545-7:1999 [29], and paving elements were tested via the Bohme abrasion test according to NBN EN 1338:2003 [30]. The aim of the performed tests was the verification of the composites’ photocatalytic performance properties after the aging tests. The abrased material was not investigated.

Assessing the risk of releasing nanometric particles from nano-modified construction materials during their lifecycle is one of the critical issues regarding further application of them. Therefore, the scientific project the authors are conducting focused at one of its stages on assessing the risk involving the mobilization of photocatalytic nanoparticles from cement matrix under abrasion load, which typically occurs during the usage of any pavement. In the paper, the authors investigated and developed laboratory methods to simulate the abrasion of pavement surfaces with a low-enough intensity to allow for an analysis of the nanoparticulate granulation of mobilized grains. The risk of photocatalytic nanoparticulate emissions due to abrasion was then investigated—three variants of cementitious photocatalytic pavement coatings, differing in material composition, were studied. The study’s goal was to determine the risk associated with emissions of nano-modifiers from the cement matrix of the pavement material due to simulated light traffic–continuous pedestrian usage. It was assumed that although photocatalytic modification of cementitious composite allows for an increase in its application potential, it must be verified through laboratory tests whether such composites contribute to a deterioration in public health when exposed to mechanical loads through an increase in the concentration of suspended dust of different granulations in the air.

For the purpose of the study, a protocol for light abrasion of cementitious composites was developed, and the size, the number of abrased grains, and their chemical composition were analyzed. As the standard abrasion resistance test on the Bhome disc might not entirely reflect the everyday conditions under which the precast elements are used, a rub abrasion test with a textile was adopted using a crockmeter.

## 2. Materials and Methods

### 2.1. Photocatalytic Cementitious Composites

Six variants of photocatalytic cementitious mortars were considered for abrasion tests, and their material composition differed. Three pairs of mortars were considered, consisting of a reference mortar without TiO_2_ and a photocatalytic mortar of the same composition (Table 1). Each photocatalytic mortar was characterized by the same mass content of nanoparticulate photocatalytic modifier (12.5 kg/m^3^) and the same water-to-cement ratio (0.36). Mortars differed in the cement-to-sand ratio, from 0.72 to 0.81. A superplasticizer was used to modify the rheological parameters of mortars and obtain similar mortar liquidity, which was determined using the slump flow method. A mini-slump cone (upper diameter = 100 mm, lower diameter = 120 mm) was used in the test, and the average from two perpendicular measurements of slump flow diameters was noted. The density of the mortars was calculated using the geometrical method.

The cement CEM I 42.5 R (Ożarów, Poland) used in the study met the requirements of EN 197-1 [31]. Its specific surface area was measured via the Blain method and equaled 3920 cm^2^/g. The micro-silica used in the study (Łaziska, Poland) met the requirements of EN 13263-1 [32] and consisted of the properties presented in [19]. The BET method measured its specific surface area, which was 23.86 m^2^/g. The quartz powder used in this study met the requirements of ISO/DIS 3262-13 [33]. The study used two types of fire-dried quartz sand aggregates of different granulations: 0.1/0.5 and 0.5/1.2 (Corrado, Poland), which met the requirements of EN 13139 [34].

**Figure 1:**
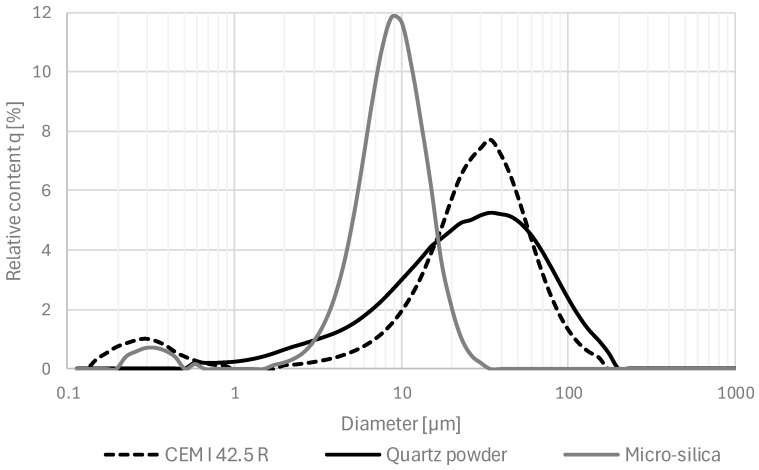
The relative granulations (q) for cement CEM 42.5 R, micro-silica, and quartz powder that were used in the study.

The cement, micro-silica, and quartz powder granulations were measured via the laser diffraction technique. The test was performed using the laser scattering method with the laser analyzer Horiba LA-300. It involved passing laser beams through an isopropyl alcohol solution containing powder grains and determining their particle diameter (in the range of 0.01–600 μm). Based on the test, granulation curves were prepared (Figure 1).

The chemical composition of cement, micro-silica, and quartz powder was investigated via the XRF method. Test samples were initially dried at 105 °C. Next, they were placed in special measuring cups and placed in the XRF apparatus, and their chemical composition was investigated. Loss of ignition (LOI) was also investigated. Powder samples were dried to a constant mass and subjected to a calcination process at 975 °C for 15 min. After cooling the samples to room temperature, their mass was determined. The percentage loss of the initial mass was the loss of ignition, which was included in the chemical composition results and is presented in Table 2.

**Figure 2:**
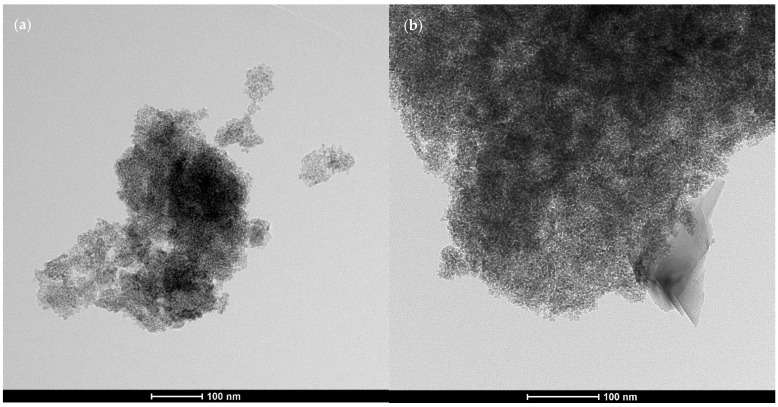
Morphology of agglomerated nanometric photocatalytic materials used in the study: TiO_2_ (A)—micrograph (**a**); TiO_2_ (B)—micrograph (**b**). TEM analysis performed on copper grids covered with a carbon film (microscope—TEM Tecnai TF 20 X-TWIN; parameters—EDAX, voltage 200 kV, STEM images collected using the HAADF detector).

Two types of nanometric titanium dioxide were used in this study: TiO_2_ (A)—K7000 (Leverkusen, Germany) and TiO_2_ (B)—P25 (Shanghai, China), with properties in the powder state as described in [35]. The content of the individual crystalline phases, the size of the crystallites, and the specific surface area of the photocatalysts are presented in Table 3. The content of the individual crystalline phases and the size of the crystallites in the tested samples were measured via the XRD method, and the specific surface area was measured via the BET method. The chemical composition (XRF) of the photocatalysts is presented in Table 2. The morphology of the grains of both TiO_2_ samples is presented in SEM images in Figure 2. Both considered modifiers exhibited photocatalytic potential in UV light, with K7000 being additionally active in the visible light spectrum.

The water used in this study met the requirements of EN 1008 [36]. The superplasticizer used in the study met the EN 934-2 [37] requirements and was characterized by electrostatic and steric mechanisms of action. Its maximal content in cementitious composite was set by the manufacturer to 3% mass of cement.

Mortars were mixed in the mixer compliant with EN 196-1 [38] using the same procedure as in the aforementioned standard. Three prismatic samples of 140 × 160 × 40 mm (a × b × h) dimensions were prepared for each series. Before demolding, the samples were stored for 24 h in the laboratory and covered with plastic foil. After demolding, the samples were cured in the curing chamber (RH > 95%, Temp = 20 +/− 2 °C) for 27 additional days. Afterward, the samples were mechanically cut to 140 × 80 × 20 mm (a × b × h), washed, and dried prior to the abrasion tests.

### 2.2. Test Setup

**Figure 3:**
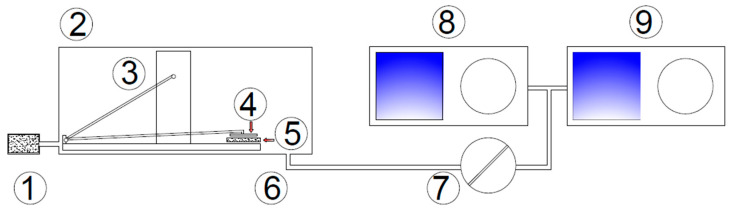
A schematic diagram of the experimental setup applied in the research of particle size analysis: (1) HEPA filter, (2) plexiglass chamber, (3) crockmeter, (4) grinding cloth, (5) tested sample, (6) air with particle inlet, (7) flow meter, (8) Scanning Mobility Particle Sizer, (9) Aerodynamic Particle Sizer.

A test setup developed by the authors at the Central Geophysical Observatory of IGF PAN in Belsk, Poland, was adopted to simulate an abrasion load of pedestrian traffic, which aimed to investigate the granulation and chemical composition of fine grains mobilized from mortar samples due to abrasion load. To achieve that goal, prepared mortar samples were placed in a sealed chamber made of plexiglass in the abrasion resistance tester crockmeter model 418 (Taber Industries, New York, NY, USA) (Figure 3). A repeated friction load was applied on the 140 × 80 mm surface of the mortar sample with a frequency of 1 Hz using the aforementioned device. A standard crocking cloth fixed to an acrylic rubbing finger resting on the sample’s surface was used. The abrasion path was set to approx. 100 mm and the vertical force transferring to the sample’s surface equaled 9.0 N. Each mortar sample was submitted to a total number of 180 cycles of abrasion.

Air flow was provided by a Thomas-type pump located at the end of the sampling path at an average rate of 4 L/min and supplied to the chamber through the HEPA filter (TSI Incorporated, Shoreview, MN, USA). The content and quality of suspended solids in the air with abrased material from the inside of the testing chamber were analyzed by two particle scanners with a different measuring range—a Scanning Mobility Particle Sizer Model 3054 (TSI Incorporated, Shoreview, MN, USA) and an Aerodynamic Particle Sizer (TSI Incorporated, Shoreview, MN, USA). The polluted air was introduced to the analyzers through an outlet located next to the abrasion platform. Additionally, a base from the SEM microscope covered with carbon tape was placed at the chamber outlet to collect the abrased material directly. The collected particles were then analyzed in terms of morphology and chemical composition.

#### 2.2.1. Abrasion Test Procedure

The test sample was placed in the crockmeter. The mortar sample was taped to the crockmeter’s table to prevent dislocation during the test. Despite using a HEPA filter to reduce any contaminants from the outside of the setup that might impact the measurement results, particles that were not the effect of sample abrasion were recorded during the tests. Particle distributions were recorded twice in the test, before and after abrasion, as background to the abrasion measurement. After a preliminary analysis of the size distribution of background particles, the results of the third background measurement after abrasion were included for further analysis.

The test procedure began after sealing the test chamber and consisted of three steps:

- Analysis of the granulation of background aerosols in the testing chamber before abrasion of the mortar sample (of origin other than the tested mortar sample);
- Analysis of the granulation of aerosols mobilized from the mortar sample due to an abrasion load;
- Analysis of the granulation of background aerosols in the testing chamber after abrasion of the mortar sample.

Each step consisted of three measurements, with each measurement lasting for three minutes. The granulation of suspended solids mobilized from the mortar surface due to an abrasion load was calculated in real time by the aforementioned set of granulation analyzers with different particle size ranges:

- 10.4 nm–469.8 nm;
- 0.523 µm–19.810 µm.

#### 2.2.2. SEM Analysis

Material for SEM analysis was collected in an additional abrasion run with a test sequence analogous to the abrasion tests. A SEM sample table was placed at the inlet pipe. On the top face of the table an adhesive carbon tape was placed, where abrased material was immobilized. Afterward, the collected samples were put in a sealed box and underwent microscopic analysis.

In the analysis, a NanoSEM 200 (FEI, Hillsboro, OR, USA) microscope with electron backscatter diffraction (EBSD) was used with an EDS detector–X-ray energy dispersive spectrometer (EDAX, Pleasanton, CA, USA). The imaging analysis determined the size of the collected abrased material and the size of individual grains. Their chemical composition was investigated with EDS.

## 3. Results and Discussion

**Figure 4:**
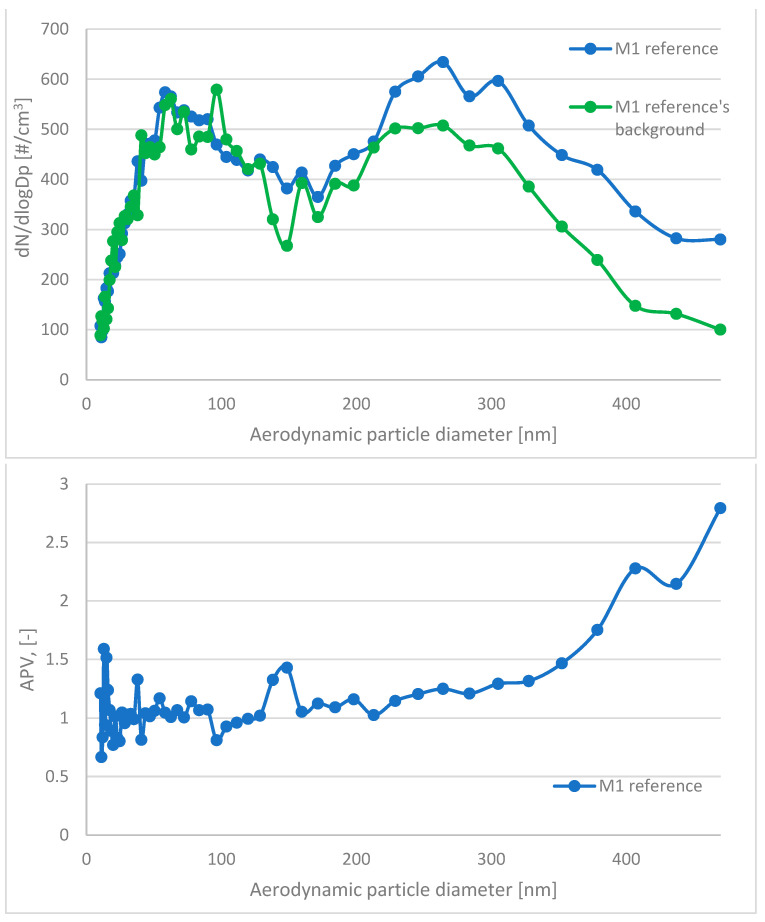
An example of a comparison of grain diameter distribution. Figure on the **top**—results of the reference’s sample abrasion (blue) vs. results background (green). Figure on the **bottom**—results presented as an abrasion parameter value (APV).

In this paper, the analyzed results refer to average values obtained in the study. The respiratory system is the most dominant ingress route of nanoparticles; therefore, in this study, the emission and size distribution of grains abrased from cementitious composite was investigated. An abrasion protocol different from those typically used in concrete technology had to be developed, as the authors wanted to investigate a particular grain emittance type. Usually, the abrasion resistance is determined through an intensive simulated load (that is the case in the Bohme test) regarding the mass loss of the sample after the procedure. However, due to the significant load present in such test conditions, mobilized grains include aggregate grains, as well as micro and millimetric grains of cement matrix. Also, the purpose of such tests is different—it is to assess the abrasion resistance of the element, not to investigate the characteristics of the mobilized material. The considered issue of the surface properties of the composite required a different approach to the test setup. To investigate the emittance potential of particles of nanometric diameters, the authors established a test procedure of much lower intensity. As a result, the outer surface of the tested samples was exposed to a low-stress abrasion, which could simulate day-to-day exposure to mechanical loads of a standard concrete pavement. Such test conditions contributed to a significantly lower number of mobilized grains and influenced their granulation. To properly assess the granulation characteristics of the mobilized particles, the authors had to consider that particles of similar characteristics constitute one of the most typical air pollutants present in the air (PM 2.5 and PM 10). Hence, to address this issue and improve the quality of the experiment, a parameter was implemented to quantify the results obtained and to assess the risk of increased particle emissions from the material into the air during abrasion: the ratio of grains recorded during the background measurement to the number of grains emitted during the abrasion test for each grain diameter (Figure 4). It was calculated based on the initial concentration of particles of considered diameters in the sealed testing chamber prior to the beginning of the abrasion test (background) and their concentration during the aforementioned test. An abrasion parameter value (APV) close to 1 indicates no additional grain emission from the sample due to abrasion (no significant change in the content of particles of the considered diameters in the measuring apparatus due to abrasion of the sample). In contrast, values greater than 1 indicate how many times the emission in a given grain diameter range increased due to applying an abrasion load on the sample’s outer surface.

The APV for the grain diameter in the range of 10.4 ÷ 469.8 nm of all investigated samples—reference and TiO_2_ ones—was similar and comparable to the aerosols’ background content of that granulation. In contrast, grain emissions differed significantly in the range of 0.523 ÷ 19.810 μm (Table 4)—the APV values for samples with nanometric TiO_2_ were in the range of 3.31 ÷ 18.42, while the values of the reference samples were in the range of 7.11 ÷ 107.18. The standard deviation of the APV for the grain diameter of 0.523÷19.810 μm for reference samples was significantly higher than that for the TiO_2_-modified samples—from 8.93 to 153.61 (reference) and from 3.53 to 28.92 (TiO_2_-modified).

**Figure 5:**
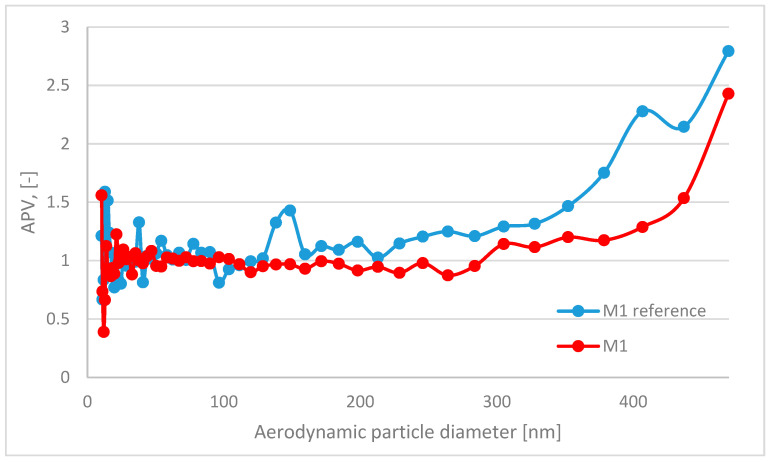
Comparison of grain diameter distribution for grains emitted during the abrasion test. APV values for mortar sample M1—reference (blue) vs. TiO_2_-modified samples (red) for grain diameters of 10.4 ÷ 469.8 nm.

**Figure 6:**
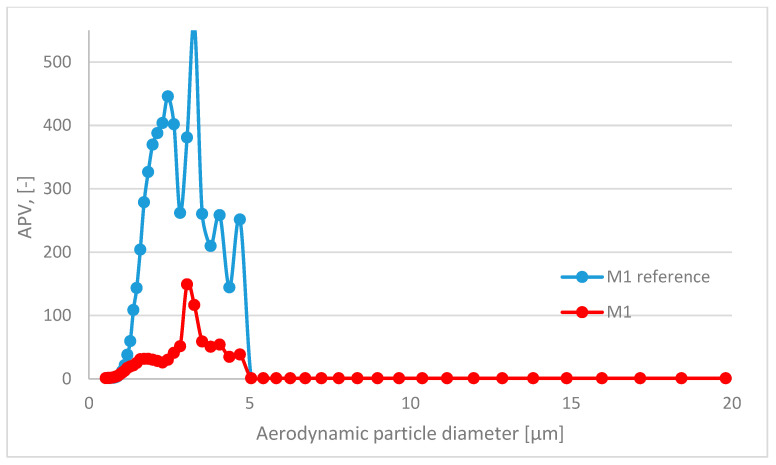
Comparison of grain diameter distribution for grains emitted during the abrasion test. APV values for mortar sample M1—reference (blue) vs. TiO_2_-modified samples (red) for grain diameters of 0.523 ÷ 19.810 μm.

**Figure 7:**
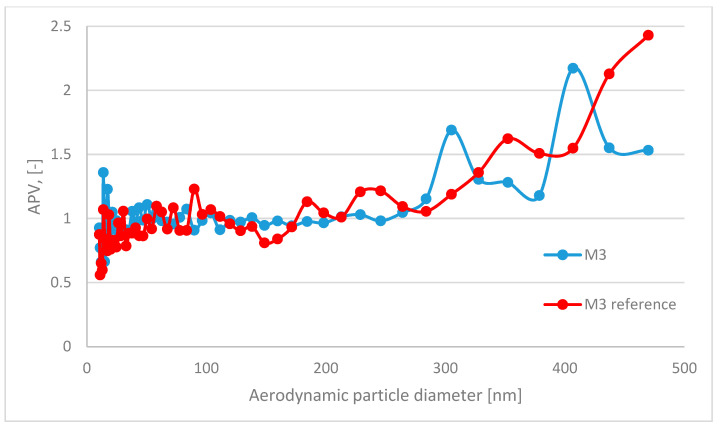
Comparison of grain diameter distribution for grains emitted during the abrasion test. APV values for mortar sample M3—reference (blue) vs. TiO_2_-modified samples (red) for grain diameters of 10.4 ÷ 469.8 nm.

**Figure 8:**
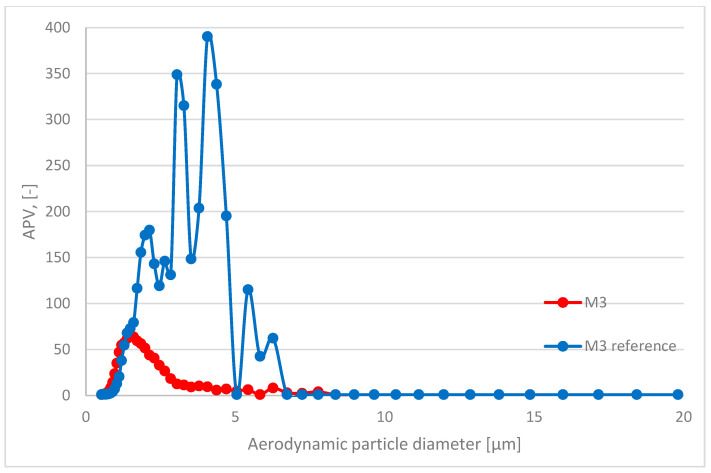
Comparison of grain diameter distribution for grains emitted during the abrasion test. APV values for mortar sample M3—reference (blue) vs. TiO_2_-modified samples (red) for grain diameters of 0.523 ÷ 19.810 μm.

A trend was observed for all analyzed sample variants, where the APV for the TiO_2_-modified samples was lower than for the reference samples. The highest emittance levels were observed for grains of approx. 1.0 to 5.0 µm, especially for reference variants (Figure 5, Figure 6, Figure 7 and Figure 8).

The nanomodification of cementitious composites allows them to acquire new functionalities or for their basic properties to be enhanced. The most common considers using nano-silica or powdered quartz to increase concrete’s mechanical performance—both focus on the densification of the cement matrix. In the case of nano-silica, pozzolanic reactions cause the formation of additional C-S-H and C-A-S-H phases, reducing the overall porosity of the cement matrix and improving its properties [39]. The introduction of powdered non-reactive powders (powdered quartz or others), although not directly involved in the hydration of the binder, contributes to a reduction in the porosity of the matrix and, therefore, increases the mechanical performance of the composite [40]. Such materials are usually referred to as micro- or nano-fillers, as they allow for the cement matrix’s densification by physically filling its micro- and nanopores. Although titanium dioxide does not have binding properties and is not treated as an additive of pozzolanic or latent hydraulic properties in concrete technology, it has been proven that its addition to a cementitious material can enhance its performance [41]. It is economically unfounded to consider the addition of TiO_2_ in significant amounts with a sole focus on the enhancement of the mechanical properties of the composite (nanoparticulate TiO_2_ is several times more expensive than any other ingredients of cementitious composites). However, an increase in the density of the cement matrix due to TiO_2_ modification is caused by the same phenomena associated with the use of other non-reactive filler powders. In the case of titanium dioxide, due to the nanometric diameters of modifier grains, it can facilitate the nucleation of hydration products over the outer surface of the nano-TiO_2_ grains, as well as reduce the porosity of the composite [42]. In the conducted research, this phenomenon of an increase in the mechanical properties of the cement matrix due to TiO_2_ modification was confirmed through indirect means—the developed abrasion test. The investigated mortars differed in the cement-to-sand ratio (the ratio between the amount of cement paste and fine aggregate filler material). The change in the aforementioned ratio increased the overall volume of the cement matrix in consecutive tested mortars (M1, M2, and M3). With the assumed force and intensity of the abrasion load in the performed tests and the test setup allowing for an analysis of the granulation of aerosols—grains fine enough to act as suspended solids in the air—only grains mobilized from the cement matrix were analyzed. As all sample series were modified with the same mass amount of nanometric TiO_2_, it was the authors’ initial intent to determine the scope of the change in the properties of the near-surface layer of the composite, with an initial premise that within the assumed scope of the variability of the cement-to-sand ratio, the effect of TiO_2_ would differ between the series and reach its peak for the series of the highest TiO_2_-to-cement mass ratio (M1). However, it was found that the effect on the properties of the near-surface layer of the composite was comparable for all titanium-modified series. With the introduction of TiO_2,_ a significant reduction in the emissions of aerosols of diameters below 5.0 µm was observed. Moreover, no additional emissions of nanometric TiO_2_ grains were detected, confirming through an indirect means the densification of the near-surface layer of the cementitious composite due to the conducted modifications.

**Figure 9:**
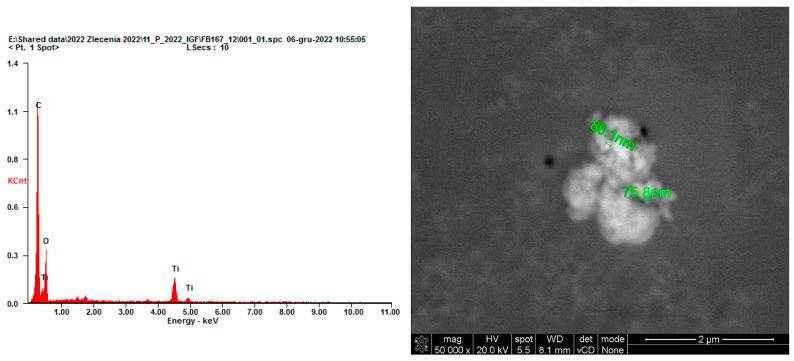
SEM micrograph of a grain of agglomerated TiO_2_ nanocrystallites mobilized from cementitious mortar (M1)—confirmation of chemical composition presented through an EDS analysis; the entire agglomerate had a diameter of approx. 1.50 µm.

As load is introduced to a cementitious system, stress and displacements propagate over the composite’s mineral skeleton. The dependence between those two values is relevant in concrete technology and accurately describes the mechanical performance of a cementitious composite. Abrasion is one of the most commonly occurring phenomena that contributes to deterioration in the performance of concrete pavement elements over time. With the introduction of an abrasion dynamic load, the outer surface of the composite is exposed to a combination of stresses (compressive, tensile, and various variants of those—shear, among others), which in value exceed the strength of the material. As a result, the propagation of numerous randomly oriented microcracks occurs, weakening the modulus of the composite, which results in an additional energy release, providing a reasonable explanation for the emission of fine particles from the surface of the composite [43]. The size of grain emittance due to such conditions depends heavily on the properties of the composite, regarding both its overall mechanical performance and chemical composition, and the intensity of the abrasion load. In the case of the conducted research, the proposed abrasion method allowed for mobilization of grains of diameters below approx. 7 µm from the outer surface of the composite. Although the number of mobilized grains from the TiO_2_-modified composites diminished significantly compared to the reference series, their chemical composition and morphology were also studied. This part of the research investigated whether solely nano-TiO_2_ grains were mobilized from the cement matrix or whether they constituted a part of agglomerated cement matrix grains (of TiO_2_ and other hydration products). As the test setup focused on obtaining grains of diameters that could contribute to an increase in PM 2.5 and PM 10 pollution (aerosols with the most harmful impact on human health), the collected samples for microscopic imaging were contaminated by fibers from cloth that was used in the abrasion test. Within that cloth, grains that were emitted from the mortar samples were immobilized. Only the TiO_2_ samples were investigated in SEM analysis, as the primary goal of the research was to determine whether photocatalytic cementitious materials contribute to an increase in aerosol concentration and quality. Throughout several attempts, only one grain consisting of only TiO_2_ was identified (Figure 9). Even in that case, crystallites of TiO_2_ agglomerated into a grain with a diameter of approx. 1.50 µm. The limited observance of such grains was probably caused by a homogenous distribution of TiO_2_ within the cement matrix.

**Figure 10:**
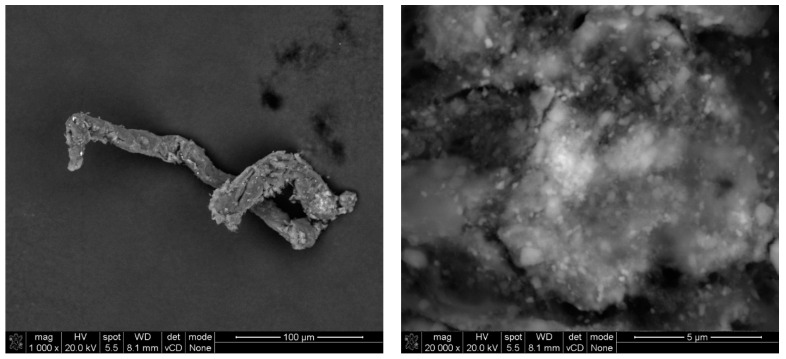
SEM micrograph of mobilized grains through an abrasion test of the mortar’s surface (M2) and immobilized on the adhesive fiber, with magnification on the immobilized material.

**Figure 11:**
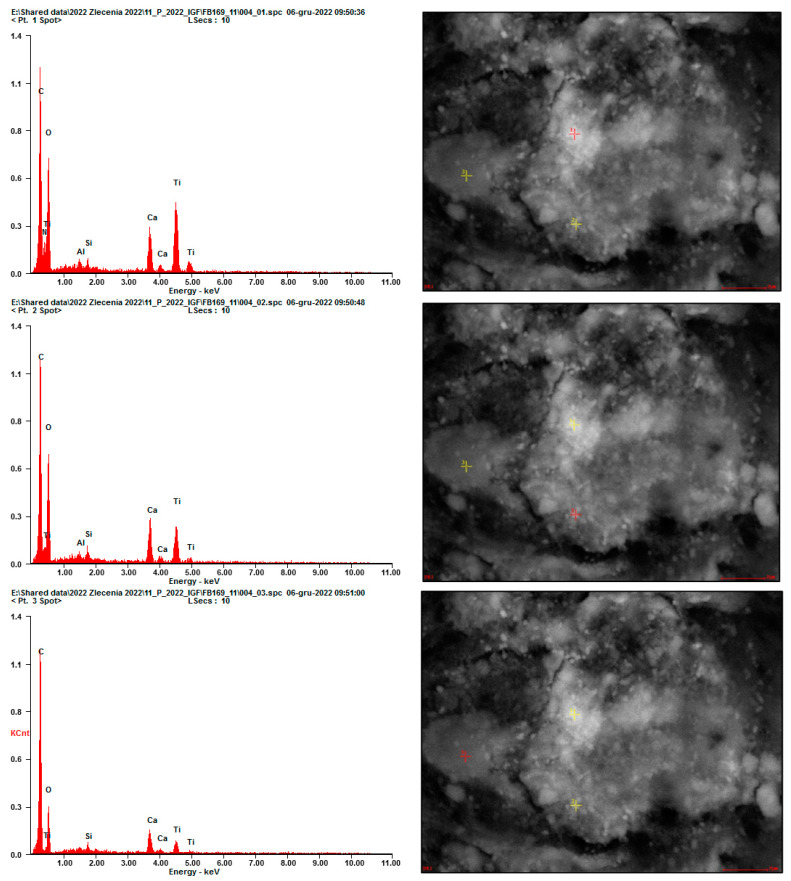
EDS analysis for different mobilized grains presented in an SEM micrograph in Figure 11; all EDS signals indicate a mix of various compounds at the exact location (red marker)—grains consisting of hydration products with TiO_2_ grains embedded within.

**Figure 12:**
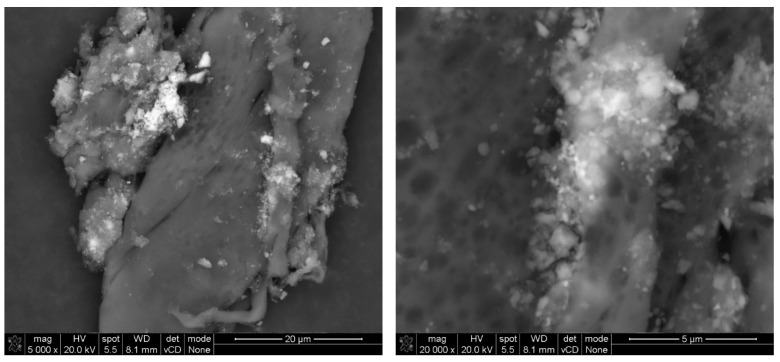
SEM micrograph of mobilized grains through an abrasion test of the mortar’s surface (M3) and immobilized on the adhesive fiber, with magnification on the immobilized material.

**Figure 13:**
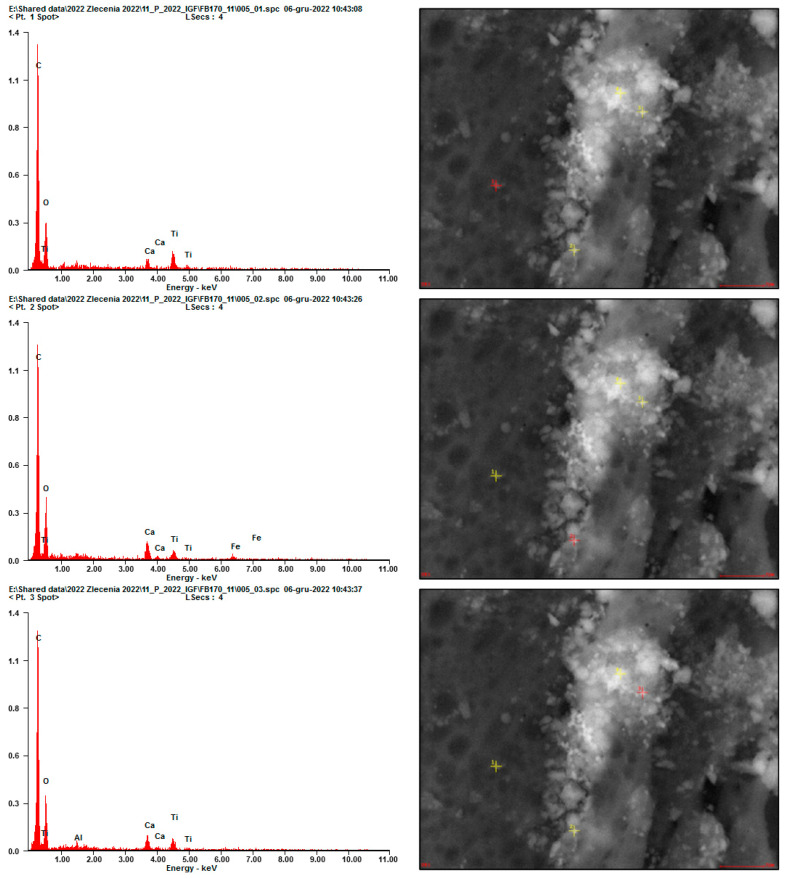
EDS analysis for different mobilized grains presented in an SEM micrograph in Figure 13; all EDS signals indicate a mix of different compounds at the exact location (red marker)—grains consisting of hydration products with TiO_2_ grains embedded within.

Finer grains were observed once immobilized on the adhesive tape fibers located at the inlet of the testing setup (Figure 10, Figure 11 and Figure 12). Due to the low intensity of the abrasion test, a small number of immobilized grains was observed. EDS analysis confirmed that the grains mainly consisted of Ca, Si, Al, and Ti elements for all considered cases, suggesting that they were of cement matrix origin—no matrix-constituent compounds mobilized independently (Figure 13). The high content of C and O presence in the EDS analysis was caused by the chemical composition of the adhesive tape on which the aerosols were immobilized.

As the nano modifier is introduced into the composite, products of various reactions between binder ingredients and water form with it a homogenous matrix during hydration. Although, as presented in Figure 2, TiO_2_ crystallites have a spherical morphology and tend to agglomerate, such agglomerates were not observed as a result of the performed abrasion tests. Knowing that nanomaterials usually exhibit different properties than their macroscopic variants (photocatalytic properties in the case of TiO_2_), the authors consider that nano-TiO_2_ could additionally act as a nucleation core for hydration products. Although such a role is beneficial regarding the mechanical performance of the composite, intensification of binder hydration contributes to TiO_2_ grains being covered with hydration products, limiting their exposure to external pollutants [44]. In the case of photocatalytic cementitious composites, a photocatalyst needs to be exposed to an external source of electromagnetic radiation of specific wavelengths for photocatalytic reactions to occur [45]—it needs to be located on the outer surface of the element. It can be said that as the quality of TiO_2_ embedment in the cement matrix increases, the photocatalytic performance of the composite is negatively affected [46]. In the case of this research, TiO_2_ was added directly to other ingredients before mixing. However, there are various other methods of applying TiO_2_ to the exposed surface of a composite—immersion in TiO_2_ solution [47], coating [48], spraying mix ingredients [49], and other types of surface modifications [25]. All of those assume that the higher the exposure of photocatalytic grains on the surface, the higher the efficiency in the photocatalytic performance of the composite. However, almost no effort is put into the durability of such solutions, especially if exposed to various environmental loads. If tested, it usually involves the investigation of a decrease in photocatalytic efficiency over time, including the development of countermeasure methods (mainly focusing on various maintenance protocols of photocatalytic surfaces) [50,51]. The mobilization potential of exposed nanometric photocatalytic grains from cementitious surfaces is usually not investigated, although it is assumed that such surfaces would be subjected to various mechanical loads.

If no additional modification is implemented, TiO_2_ grains distribute homogenously throughout the volume of the cement matrix as grains of agglomerated crystallites of diameters dependent on the composite preparation protocol. In the case of this study, TiO_2_ constituted approx. 1% of the volume of the cement matrix—the same ratio could be assumed regarding its presence on the outer surface on which abrasion was investigated.

The conducted test verified the proper embedment of the nano modifier in the cement matrix, confirming its compatibility with the cement matrix (Figure 12 and Figure 13). Although exposed to an external load, TiO_2_ grains contributed to increased resistance of the near-surface layer of the composite, suggesting its influence over cement matrix organization and structure. The effect of photocatalytic modifications is usually investigated in terms of the ability of the composite to either decompose gaseous pollutants [52] (organic and inorganic—nitrogen oxides, ozone, aromatic hydrocarbons, and others) or through self-cleaning tests (decomposition of different organic dyes under exposure to electromagnetic radiation of a specific wavelength and irradiation—the rhodamine b test, for instance [19]). Those test procedures assume verification of the surface properties—the quality and the concentration of TiO_2_ embedded in the outer surface of the element. With an increase in the TiO_2_ content in that layer, the overall photocatalytic performance of the composite is enhanced. However, those methods do not provide any information regarding the quality of TiO_2_ immobilization within the cement matrix. The proposed light abrasion test for photocatalytic composites allowed us to determine whether the modifier’s grains were properly embedded in the cement matrix and did not pose a significant mobilization risk during exposure to light mechanical loads.

## 4. Conclusions

Throughout the conducted research, the authors confirmed that in the developed laboratory conditions, due to the nanometric diameter of TiO_2_ grains, the modifier contributed to an enhancement in the near-surface mechanical performance of the hardened composite, preventing excessive nanometric emissions due to an abrasion load. Therefore, the cement matrix constituted an environment for stable TiO_2_ immobilization, allowing it to contribute to various photocatalytic reactions without a significant risk of increasing the concentration of harmful airborne aerosols under mechanical load. The release of grains of nano-metric diameters during abrasion was not observed. Although a grain composed only of TiO_2_ was found in SEM/EDS analysis, it had agglomerated to a size of up to 2.0 μm, which was the average size of abrased grains observed in the research. Based on the above observations, it should be concluded that the risk of the release of nanometric particles during the abrasion process is minimal.

## Figures and Tables

**Table 1:** The composition and basic properties of tested mortars.

Component/Series ID	M1	M2	M3	M1 (Ref)	M2 (Ref)	M3 (Ref)
	**Content (kg/m^3^)**					
Cement	794	812	837	794	812	837
Silica	10	10	10	10	10	10
Quartz powder	89	87	84	89	87	84
Water	286	292	301	286	292	301
Sand 0.1/0.5	424	413	397	424	413	397
Sand 0.5/1.2	679	661	635	679	661	635
TiO_2_ (A)	2.5	2.5	2.5	-	-	-
TiO_2_ (B)	10	10	10	-	-	-
Superplasticizer	4.76	4.87	5.02	4.21	4.54	4.89
Mortar properties						
w/c *	0.36	0.36	0.36	0.36	0.36	0.36
c/s **	0.72	0.76	0.81	0.72	0.76	0.81
Slump flow, mm	320	310	315	315	315	320
Density, kg/m^3^	2245	2235	2220	2240	2235	2225

* Water-to-cement ratio (mass), ** cement-to-sand ratio (mass).

**Table 2:** Chemical composition (XRF) of cement, nanoparticulate silica, quartz powder, and photocatalysts used in the study.

Material	Oxide (wt. %)									LOI, %
	CaO	SiO_2_	Al_2_O_3_	MgO	SO_3_	TiO_2_	P_2_O_5_	Fe_2_O_3_	MnO	
Cement	67.2	16.8	3.3	0.9	4.0	0.3	0.2	3.8	0.2	2.3
Silica	0.4	96.7	1.3	<0.1	-	<0.1	<0.01	0.2	<0.01	1.1
Quartz powder	0.1	94.3	0.2	1.2	-	<0.1	0.1	0.6	0.1	2.5
TiO_2_ (A)	-	0.2	-	-	0.4	99.2	0.06	-	-	n.d. *
TiO_2_ (B)	-	0.7	-	-	-	99.1	-	-	-	n.d. *

* n.d.—Not determined.

**Table 3:** Properties of photocatalysts used in the study (SSA—specific surface area).

Photocatalyst	Phase Composition (%)		Size of Crystallites (nm)		Wettability	SSA (m^2^/g)
	Rutile	Anatase	Rutile	Anatase		
TiO_2_ (A)	-	100	-	10	Hydrophilic	246.8 +/− 2.9
TiO_2_ (B)	13	87	54	33	Hydrophobic	53.8 +/− 0.2

**Table 4:** APV for investigated cement mortar samples.

	APV for Grain Diameter Range 10.4 ÷ 469.8 nm	Standard Deviation for APV in Range 10.4 ÷ 469.8 nm	APV for Grain Diameter Range 0.523 ÷ 19.810 μm	Standard Deviation for APV in Range 0.523 ÷ 19.810 μm
M1	1.03	0.26	18.42	28.92
M1 (ref)	1.17	0.37	107.18	153.61
M2	0.98	0.17	3.31	3.53
M2 (ref)	1.08	0.14	7.11	8.93
M3	1.04	0.24	17.01	21.43
M3 (ref)	1.03	0.33	71.40	102.75

## Data Availability

The data are contained within the article.
